# ﻿One new genus and four new species of Liocranidae Simon, 1897 (Arachnida, Araneae) from China and Vietnam

**DOI:** 10.3897/zookeys.1181.108822

**Published:** 2023-10-06

**Authors:** Chang Chu, Shuqiang Li, Yanbin Yao, Zhiyuan Yao

**Affiliations:** 1 College of Life Science, Shenyang Normal University, Shenyang 110034, Liaoning, China Shenyang Normal University Shenyang China; 2 Institute of Zoology, Chinese Academy of Sciences, Beijing 100101, China Institute of Zoology, Chinese Academy of Sciences Beijing China; 3 Jinshan College of Fujian Agriculture and Forestry University, Fuzhou 350002, Fujian, China Forestry University Fuzhou China

**Keywords:** Biodiversity, morphology, new taxa, Southeast Asia, spiny-legged sac spiders, taxonomy

## Abstract

Four new species of the family Liocranidae are described from China and Vietnam. The new genus *Sinocranum***gen. nov.**, is erected to accommodate *S.menghai***sp. nov.** (♂♀) from China. Further new species described include *Koppeninger***sp. nov.** (♀) from China, *Xanthariabaizilongi***sp. nov.** (♂♀) from China and *X.cucphuong***sp. nov.** (♂) from Vietnam. In addition, *Xantharia* is transferred from Miturgidae to Liocranidae. *Koppe* and *Xantharia* are reported from China and Vietnam, respectively, for the first time.

## ﻿Introduction

Liocranidae Simon, 1897, also known as spiny-legged sac spiders, are free-living, ground-dwelling hunters (Gündüz and Allahverdi 2018; Dippenaar-Schoeman et al. 2021; Lu et al. 2023), currently containing 35 genera and 338 species (WSC 2023). Liocranid spiders are distributed worldwide and live in a variety of ecosystems, including forests, intertidal zones, savannas, grasslands and even in desert regions (Dippenaar-Schoeman et al. 2021; Chu et al. 2023). They are small to medium-sized spiders, whose body length ranges from 3 to 15 mm, with a highly variable habitus (Jocqué and Dippenaar-Schoeman 2006; Dippenaar-Schoeman et al. 2021). The family has no clear synapomorphies, and some genera are frequently moved, including changes between Clubionidae Wagner, 1887 and Miturgidae Simon, 1886 (Marusik and Fomichev 2020; Zamani and Marusik 2021; Bosselaers and Jocqué 2022; WSC 2023). However, the characteristic of “posterior median eye tapeta forming 90° angle” can distinguish Liocranidae from Clubionidae and Miturgidae (Ramírez 2014).

*Xantharia* Deeleman-Reinhold, 2001 was initially placed in Clubionidae within the Systariinae Deeleman-Reinhold, 2001 (Deeleman-Reinhold 2001). It is currently listed in this subfamily and provisionally kept in Miturgidae (Ramírez 2014; WSC 2023). The genus currently contains three species, all distributed in Southeast Asia, but *Xantharia* has not been found in Vietnam (WSC 2023).

Deeleman-Reinhold (2001) described the genus *Koppe* based on spiders of both sexes from Indonesia described as the type species *K.montana* Deeleman-Reinhold, 2001. *Koppe* was initially placed in Corinnidae Karsch, 1880 (Deeleman-Reinhold 2001) and later transferred to Liocranidae (Ramírez 2014). The genus currently contains 14 species and is mainly distributed in South and Southeast Asia; hitherto, *Koppe* has not been found in China (WSC 2023).

The goals of the present paper are the description of one new genus, *Sinocranum* gen. nov., and four new species, *K.ninger* sp. nov., *S.menghai* sp. nov., *X.baizilongi* sp. nov. and *X.cucphuong* sp. nov., as well as the transfer of the genus *Xantharia* from Miturgidae to Liocranidae.

## ﻿Material and methods

**Figure 1.**
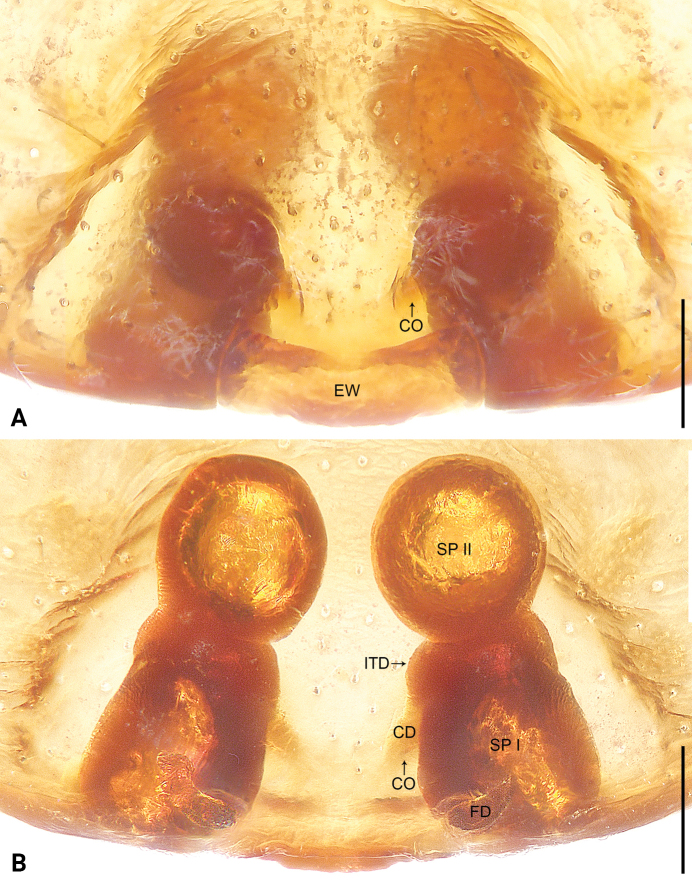
*Koppeninger* sp. nov., holotype female (**A, B**) **A** epigyne, ventral view **B** vulva, dorsal view. Abbreviations: CD = copulatory duct, CO = copulatory opening, EW = epigynal window, FD = fertilization duct, ITD = intertubular duct, SP I= spermatheca I, SP II= spermatheca II. Scale bars: 0.10 mm.

Specimens were examined and measured with a Leica M205 C stereomicroscope. Left male palps were photographed. Epigynes were photographed. Vulvae were treated in a warm 10% potassium hydroxide (KOH) solution to dissolve soft tissues before illustration. Images were captured with a Canon EOS 750D wide zoom digital camera (24.2 megapixels) mounted on the stereomicroscope mentioned above, and assembled using Helicon Focus v.3.10.3 image stacking software (Khmelik et al. 2005). All measurements are given in millimeters (mm). Palp and leg measurements are shown as palp total length (femur, patella, tibia, -, tarsus), or leg total length (femur, patella, tibia, metatarsus, tarsus). Leg segments were measured on their dorsal side. The intertubular ducts are tubes that connect primary spermathecae and secondary spermathecae; they may be very short or rather long (e.g., fig. 395 in Deeleman-Reinhold 2001; figs 1J, 2B in Sankaran 2022). The species distribution map was generated with ArcGIS 10.2 (ESRI Incorporated Company). The specimens studied are preserved in 75% ethanol and deposited in the Institute of Zoology, Chinese Academy of Sciences (IZCAS) in Beijing, China.

Terminology and taxonomic descriptions follow Deeleman-Reinhold (2001), Sankaran (2022), Chu et al. (2023) and Lu et al. (2023).

The following abbreviations are used in the descriptions:

**AER** anterior eye row;

**ALE** anterior lateral eye;

**AME** anterior median eye;

**do** dorsal;

**PER** posterior eye row;

**pl** prolateral;

**PLE** posterior lateral eye;

**plv** prolateral ventral;

**rl** retrolateral;

**rlv** retrolateral ventral;

**v** ventral.

## ﻿Taxonomy

### ﻿Family Liocranidae Simon, 1897

#### ﻿Genus Koppe

Taxon classificationAnimaliaAraneaeLiocranidae

Deeleman-Reinhold, 2001

74CB2B4D-1595-57DA-AA91-D3C76A6329E1

##### Type species.

*Koppemontana* Deeleman-Reinhold, 2001 from Indonesia.

##### Comments.

**Figure 2.**
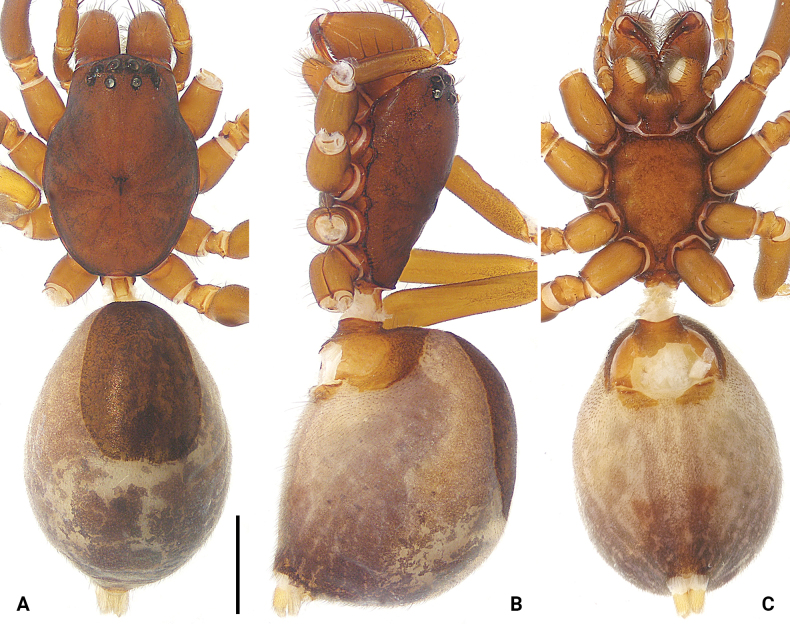
*Koppeninger* sp. nov., holotype female (**A–C)** habitus **A** dorsal view **B** lateral view **C** ventral view. Scale bar: 1.00 mm.

*Koppe* resembles *Oedignatha* Thorell, 1881 (cf. Figs 1, 2 and Deeleman-Reinhold 2001: 261, figs 348–374; Chu et al. 2023: 178, fig. 2A–D) by having massive chelicerae (Fig. 2A–C) and a simplistic genitalic organ structure (Fig. 1A), but can be distinguished by the carapace surface without granules or pits (Fig. 2A, B; present in *Oedignatha*), by the clypeus with a slight conical hump or absent (Fig. 2A; vs. clypeus with a distinct conical hump in *Oedignatha*), and by the intercoxal sclerites enlarged (Fig. 2B; absent in *Oedignatha*).

##### Composition.

*Koppe* includes 14 species distributed in Asia and Oceania. Of these, 11 species are distributed in Southeast Asia: *K.baerti* Deeleman-Reinhold, 2001 (♂♀) from Indonesia, *K.calciphila* Deeleman-Reinhold, 2001 (♂♀) from Indonesia, *K.doleschalli* Deeleman-Reinhold, 2001 (♂♀) from Indonesia, *K.kinabalensis* Deeleman-Reinhold, 2001 (♂♀) from Malaysia, *K.kuntneri* Deeleman-Reinhold, 2001 (♂♀) from Indonesia, *K.minuta* Deeleman-Reinhold, 2001 (♂♀) from Indonesia, *K.montana* Deeleman-Reinhold, 2001 (♂♀) from Indonesia, *K.no* Deeleman-Reinhold, 2001 (♂♀) from Indonesia, *K.princeps* Deeleman-Reinhold, 2001 (♂♀) from Indonesia, *K.sumba* Deeleman-Reinhold, 2001 (♂♀) from Indonesia and *K.tinikitkita* (Barrion & Litsinger, 1995) (♀) from Philippines.

#### ﻿ Koppe ninger

Taxon classificationAnimaliaAraneaeLiocranidae

Chu & Li sp. nov.

1692FDEC-1E8D-592D-A2D6-1A6828B54BD1

https://zoobank.org/3D1D9CAA-0F7B-4BEC-845E-BE9F5C67CB9D

Figs 1 , 2

##### Type material.

***Holotype***: 1♀ (IZCAS-Ar44617), **China**, Yunnan, Pu’er, Ning’er County, Jinpaoshan Park, 23°3.658′N, 101°3.466′E, hand catch in leaf litter, 26 July 2022, F. Gao leg.

##### Etymology.

The specific name refers to the type locality and is a noun in apposition.

##### Diagnosis.

The new species resembles *K.princeps* Deeleman-Reinhold, 2001 (cf. Figs 1, 2 and Deeleman-Reinhold 2001: 283, figs 391–396) by the similar rectangular epigynal window (Fig. 1A), posteriorly located copulatory openings (Fig. 1A), thin copulatory ducts (Fig. 1B) and globular secondary spermathecae (Fig. 1B). Females can be distinguished by the epigyne with a pair of long, oblique sclerotized area laterally (Fig. 1A; vs. epigyne with a pair of short, similar point-shaped sclerotized area laterally), by the intertubular ducts globular (Fig. 1B; vs. intertubular ducts tubular), by the primary spermathecae elliptical, separated by about their diameter (Fig. 1B; vs. primary spermathecae kidney-shaped, separated by more than twice their diameter), by the secondary spermathecae separated by less than half of their diameter (Fig. 1B; vs. secondary spermathecae separated by more than their diameter), and by the fertilization ducts pointing antero-laterally (Fig. 1B; vs. fertilization ducts pointing postero-laterally). This species also resembles *K.fusca* Sankaran, 2022 (cf. Figs 1, 2 and Sankaran 2022: 438, figs 1, 2) by the similar rectangular epigynal window (Fig. 1A), posteriorly located copulatory openings (Fig. 1A), thin copulatory ducts (Fig. 1B), globular secondary spermathecae (Fig. 1B) and antero-laterally pointed fertilization ducts (Fig. 1B). Females can be distinguished by the epigyne with a pair of long, oblique sclerotized area laterally (Fig. 1A; absent), by the epigyne without distinct median flap (Fig. 1A; present), by the intertubular ducts globular (Fig. 1B; vs. intertubular ducts tubular, with anterior twist), by the primary spermathecae elliptical, separated by about their diameter (Fig. 1B; vs. primary spermathecae kidney-shaped, separated by more than their diameter), and by the secondary spermathecae separated by less than half of their diameter (Fig. 1B; vs. secondary spermathecae connected to each other). Male unknown.

##### Description.

**Female** (holotype; Fig. 2A–C). Total body length 5.35, carapace 2.22 long, 1.60 wide; opisthosoma 3.13 long, 2.11 wide. Eye sizes and interdistances: AME 0.09, ALE 0.08, PME 0.09, PLE 0.08; AME–AME 0.11, AME–ALE 0.10, PME–PME 0.18, PME–PLE 0.17, AME–PME 0.12, ALE–PLE 0.09. Carapace reddish-brown, smooth, with distinct radial grooves; fovea longitudinal, slit-like. Chelicerae reddish-brown, massive, with several setae on anterior surface, with three promarginal and seven retromarginal teeth. Endites and labium reddish-brown; endites narrower in middle, subapically with large, semicircular membranous area and dense scopula; labium longer than wide, with subbasal constriction and sparse scopula apically. Sternum reddish-brown, shield-shaped, with intercoxal sclerites between coxae; posterior margin extending between coxae IV; intercoxal sclerites distinctly enlarged, especially between coxae I and II, II and III. Legs yellowish-brown. Leg spination: femur I pl 1; tibiae I plv 9 rlv 8, II plv 7 rlv 6; metatarsi I plv 6 rlv 6, II plv 5 rlv 4. Palp and leg measurements: palp 2.97 (0.92, 0.43, 0.62, -, 1.00), I 8.76 (2.10, 0.69, 2.36, 2.23, 1.38), II 6.78 (1.84, 0.62, 1.61, 1.64, 1.07), III 5.89 (1.56, 0.60, 1.16, 1.59, 0.98), IV 8.29 (2.10, 0.70, 1.87, 2.37, 1.25). Leg formula: 1423. Dorsal opisthosoma brown with grey patterns, oval, with scutum covering half of dorsum surface. Lateral opisthosoma with pale stripes. Ventral opisthosoma yellowish with brown patterns posteriorly, epigastric scutum reddish-brown. Spinnerets yellowish.

***Epigyne*** (Fig. 1A, B). Epigynal field nearly fan-shaped, with a pair of long, oblique sclerotized area laterally; posterior part medially with weakly sclerotized epigynal window. Copulatory openings hidden under epigynal plate. Copulatory ducts thin. Intertubular ducts globular. Primary spermathecae elliptical, separated by about their diameter; secondary spermathecae globular, separated by less than half of their diameter. Fertilization ducts pointing antero-laterally.

##### Distribution.

China (Yunnan, type locality; Fig. 12).

#### ﻿Genus Sinocranum

Taxon classificationAnimaliaAraneaeLiocranidae

Chu & Li gen. nov.

C8721F93-FE41-5250-9826-1C164926DD94

https://zoobank.org/05E6DFD1-0646-4FDD-9049-7D3428A31744

##### Type species.

*Sinocranummenghai* Chu & Li, sp. nov.

##### Composition.

Monotypic.

##### Etymology.

The generic name is a combination of “*sino*”, referring to the China, and “*cranum*” as part of the genus *Liocranum*. Gender is neuter.

##### Diagnosis.

**Figure 3.**
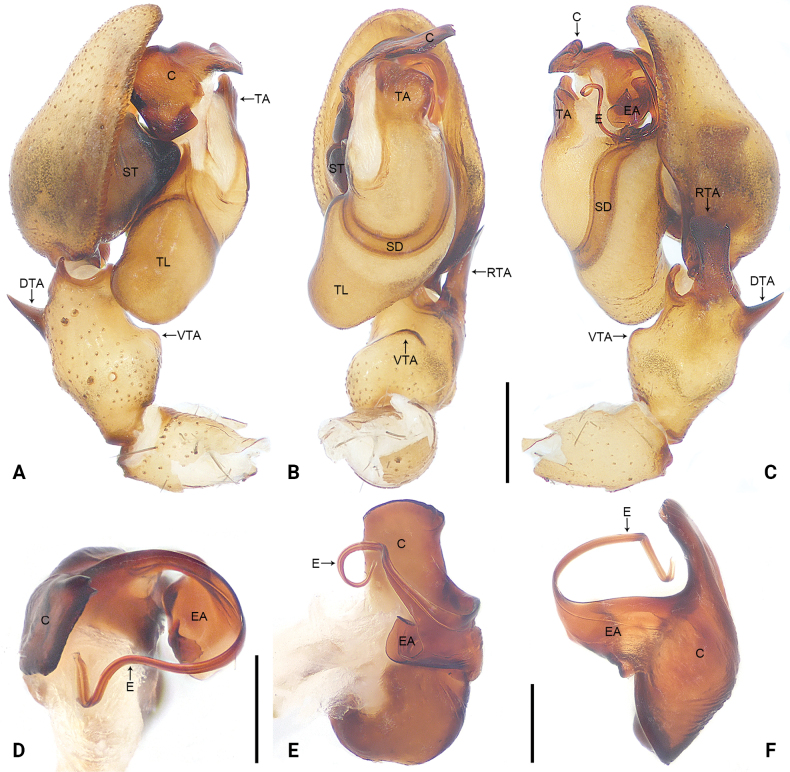
*Sinocranummenghai* sp. nov., holotype male palp (**A–C)**, conductor and embolus (**D–F) A** prolateral view **B** ventral view **C** retrolateral view **D** frontal view **E** retrolateral view **F** dorsal view. Abbreviations: C = conductor, DTA = dorsal tibial apophysis, E = embolus, EA = embolic apophysis, RTA = retrolateral tibial apophysis, SD = sperm duct, ST = subtegulum, TA = tegular apophysis, TL = tegular lobe, VTA = ventral tibial apophysis. Scale bars: 0.50 mm (**A–C**); 0.20 mm (**D–F**).

**Figure 4.**
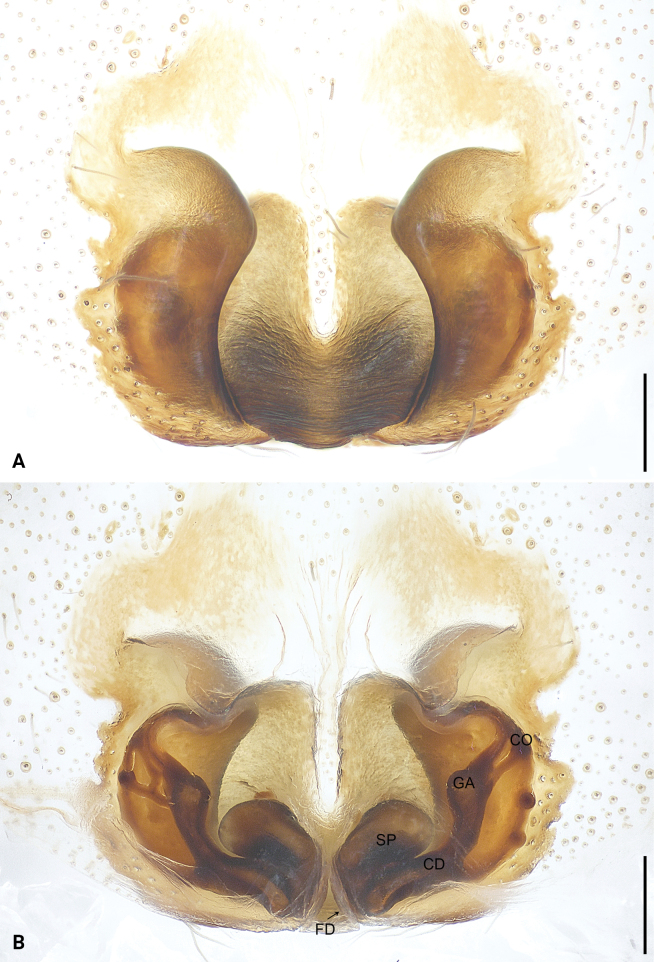
*Sinocranummenghai* sp. nov., paratype female (**A, B**) **A** epigyne, ventral view **B** vulva, dorsal view. Abbreviations: CD = copulatory duct, CO = copulatory opening, FD = fertilization duct, GA = glandular appendage, SP = spermathecae. Scale bars: 0.20 mm.

The new genus resembles *Agroeca* Westring, 1861 by having a similar tegular lobe (Fig. 3A–C) in the male and similar long copulatory ducts (Fig. 4B) in the female. Males can be distinguished by the embolus originating retrolaterally (Fig. 3C; vs. embolus originating prolaterally in *Agroeca*), by the conductor strongly sclerotized (Fig. 3A–F; vs. conductor membranous in *Agroeca*), and by the palp with ventral tibial apophysis and dorsal tibial apophysis (Fig. 3A–C; absent in *Agroeca*). Females can be distinguished by the epigynal plate without hoods (Fig. 4A; present in *Agroeca*), by the vulva with glandular appendages (Fig. 4B; absent in *Agroeca*), and by the fertilization ducts pointing anteriorly (Fig. 4B; vs. fertilization ducts pointing laterally in *Agroeca*).

*Sinocranum* gen. nov. shares several synapomorphies with the members of the genus *Agroeca*. The most important of these is tegular lobe with a distinct curved prolaterally in ventral view. Similarities can be observed in the general structure of the male palp (shape of cymbium and tibia; position of conductor and tegular apophysis). The distribution of the eyes is also similar to that in *Agroeca*. However, despite these similarities, there are still significant differences between *Sinocranum* and *Agroeca* (refer to above genus diagnosis for details). The new genus *Sinocranum* with two most obvious morphological characteristics: male palp with ventral tibial apophysis, retrolateral tibial apophysis and dorsal tibial apophysis; female copulatory ducts bifurcate from subdistally to distally. These two characteristics are different from all other existing genera in the family. Therefore, based on the above morphological data, we suggest establishing a new genus *Sinocranum* to accommodate *S.menghai* sp. nov.

##### Description.

**Figure 5.**
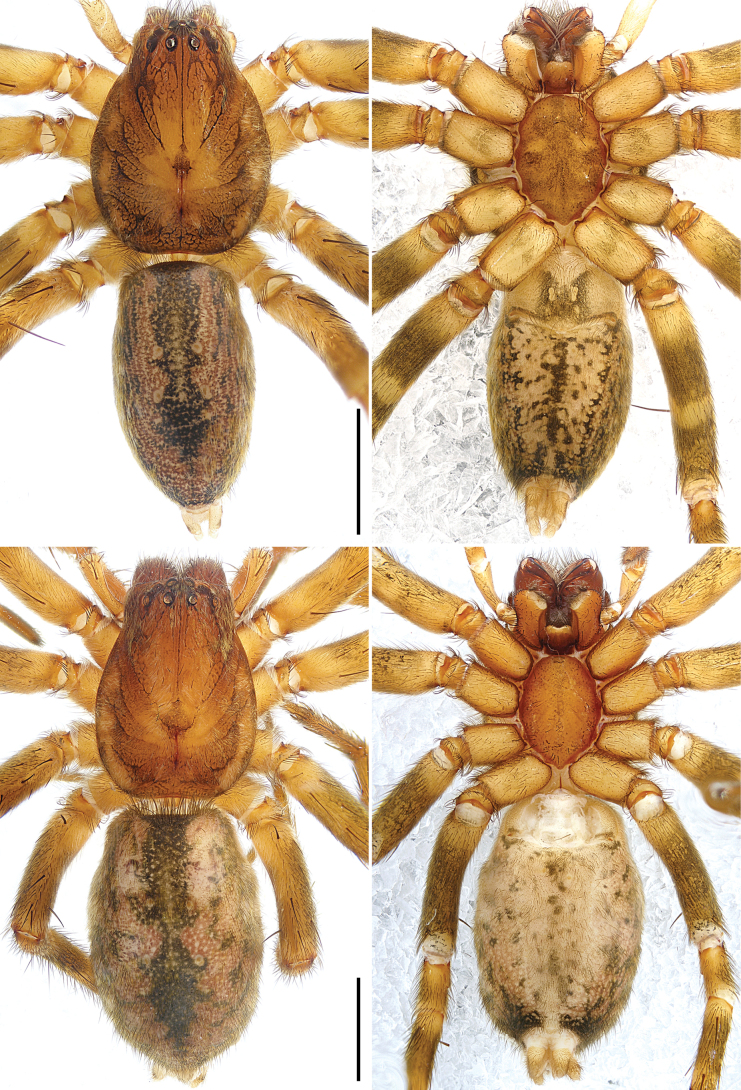
*Sinocranummenghai* sp. nov., holotype male (**A, B**) and paratype female (**C, D**) **A–D** habitus **A** dorsal view **B** ventral view **C** dorsal view **D** ventral view. Scale bars: 2.00 mm.

Small to medium-sized spiders (total body length 7.89–10.70; Figs 5A–D, 11A). Eight eyes in two rows; PER longer than AER, AER recurved, PER almost straight in dorsal view. AME separated by less than their diameter, closer to ALE; PME separated by almost twice their diameter, about as far from ALE; Distance between AME and PME longer than that between ALE and PLE; ALE and PLE separated by about their diameter. Carapace reddish-brown with lighter heart region, laterally with dark stripes, submarginally with lighter patches, marginally dark, with white hairs; fovea reddish-brown. Chelicerae reddish-brown, with three promarginal and two retromarginal teeth. Endites yellowish- to reddish-brown, longer than wide, narrower in middle, subapically with semicircular membranous area and dense scopula. Labium reddish-brown with lighter distal lip. Sternum reddish-brown. Legs yellowish-brown, lateral tarsi and metatarsi I–II with dense scopulae. Leg spination: femora with 2–4 pairs of lateral spines and 3 dorsal spines; tibiae with 0–3 pairs of lateral spines, 0–3 dorsal spines and 3 pairs of ventral spines; metatarsi with 0–4 pairs of lateral spines, 0–1 dorsal spine and 2 ventral spines or 1–3 pairs of ventral spines. Leg formula: 4123. Dorsal opisthosoma yellowish, median field with dark bands, laterally with reddish-brown stripes and dark patches. Lateral and ventral opisthosoma yellowish with dark spots and dark ring around spinnerets. Spinnerets yellowish.

Palpal (Fig. 3A–F) tibia with three apophyses; ventral tibial apophysis arc-shaped; retrolateral tibial apophysis long, with blunt and thin sheet-shaped tip; dorsal tibial apophysis straight, with wide base and narrow tip deviating from the direction of palp. Bulb longer than wide, tegular lobe curved prolaterally. Tegulum with distinct, U-shaped sperm duct subcentrally; subtegulum strongly sclerotized, clearly visible in ventral view. Embolus originating retrolaterally, long and filiform, connected to conductor. Conductor strongly sclerotized, originating distally. Tegular apophysis originating subdistally.

Epigynal field (Fig. 4A, B) with two large spots; epigynal plate sclerotized. Copulatory openings hidden under epigynal plate. Copulatory ducts long and curved. Glandular appendages globular, originating subdistally to copulatory ducts. Spermathecae large, separated by less than half of their diameter. Fertilization ducts almost as long as diameter of spermathecae, close to each other distally, pointing anteriorly.

##### Distribution.

China (Yunnan; Fig. 12).

#### ﻿ Sinocranum menghai

Taxon classificationAnimaliaAraneaeLiocranidae

Chu & Li sp. nov.

4D8A5CEA-7B79-5017-95D1-6E177CC8F402

https://zoobank.org/611BECC7-D402-4086-ABA5-19B4B435F878

Figs 3 , 4 , 5 , 11A

##### Type material.

***Holotype***: 1♂ (IZCAS-Ar44618), **China**, Yunnan, Xishuangbanna, Menghai County, Menghai Town, Man’ao Village Committee, 21°57.620′N, 100°28.261′E, 1176 m, hand catch in leaf litter, November 2022, H. Qiu leg. ***Paratypes***: 2♀ (IZCAS-Ar44619, 44620), same data as holotype.

##### Etymology.

The specific name refers to the type locality and is a noun in apposition.

##### Diagnosis.

The new species can be distinguished by the tegular lobe large and curved prolaterally (Fig. 3A–C), by the embolus originating retrolaterally, embolic apophysis coiled and thin sheet-shaped (Fig. 3C–F), by the conductor strongly sclerotized (Fig. 3A–F), by the palp with ventral tibial apophysis, retrolateral tibial apophysis and dorsal tibial apophysis (Fig. 3A–C), by the epigynal plate sclerotized, anteriorly to medially with narrow membranous area, laterally with large crescent-shaped sclerites (Fig. 4A), by the vulva with glandular appendages, originating subdistally to copulatory ducts (Fig. 4B), by the copulatory ducts long and curved, subdistally to distally bifurcate (Fig. 4B), and by the fertilization ducts almost as long as diameter of spermathecae, close to each other distally, pointing anteriorly (Fig. 4B).

##### Description.

**Male** (holotype; Figs 5A, B, 11A). Total body length 7.89, carapace 3.77 long, 2.87 wide, opisthosoma 4.12 long, 2.35 wide. Eye sizes and interdistances: AME 0.16, ALE 0.15, PME 0.14, PLE 0.17; AME–AME 0.13, AME–ALE 0.09, PME–PME 0.27, PME–PLE 0.25, AME–PME 0.20, ALE–PLE 0.16. Carapace reddish-brown with lighter heart region, laterally with dark stripes, submarginally with lighter patches, marginally dark, with white hairs; fovea reddish-brown. Chelicerae reddish-brown, with several setae on anterior surface, with three promarginal and two retromarginal teeth. Endites yellowish-brown, longer than wide, narrower in middle, subapically with semicircular membranous area and dense scopula. Labium reddish-brown with lighter distal lip. Sternum reddish-brown. Legs yellowish-brown, lateral tarsi and metatarsi I–II with dense scopulae. Leg spination: femora I pl 3 do 3 rl 3, II pl 4 do 3 rl 4, III–IV pl 3 do 3 rl 3; tibiae I pl 3 rl 3 plv 3 rlv 3, II–IV pl 3 do 3 rl 3 plv 3 rlv 3; metatarsi I–II pl 1 rl 1 v 2, III–IV pl 4 do 1 rl 4 plv 3 rlv 3. Palp and leg measurements: palp 4.95 (1.55, 0.94, 1.00, -, 1.46), I 12.09 (3.18, 1.70, 2.96, 2.63, 1.62), II 12.02 (3.30, 1.60, 2.89, 2.74, 1.49), III 10.64 (3.01, 1.39, 2.22, 2.75, 1.27), IV 13.79 (3.67, 1.56, 3.08, 3.91, 1.57). Leg formula: 4123. Dorsal opisthosoma yellowish, median field with dark bands, laterally with reddish-brown stripes and dark patches. Lateral and ventral opisthosoma yellowish with dark spots and dark ring around spinnerets. Spinnerets yellowish.

***Palp*** (Fig. 3A–F). Tibia with three apophyses; ventral tibial apophysis arc-shaped, weakly sclerotized; retrolateral tibial apophysis wide, with slight retrolateral curvature distally, with blunt and thin sheet-shaped tip; dorsal tibial apophysis straight, spine-shaped, with wide base and narrow tip deviating from the direction of palp. Bulb longer than wide, tegular lobe distinct curved prolaterally. Tegulum membranous antero-laterally, with distinct, U-shaped sperm duct subcentrally; subtegulum strongly sclerotized, clearly visible in ventral view. Embolus originating retrolaterally, long and filiform, connected to conductor; embolic apophysis coiled and thin sheet-shaped; embolus and embolic apophysis almost invisible in ventral view. Conductor strongly sclerotized, originating 10:30–11:30 o’clock. Tegular apophysis originating subdistally to bulb, nearly rectangular.

**Female** (paratype; Fig. 5C, D). Total body length 9.85, carapace 4.31 long, 3.13 wide, opisthosoma 5.54 long, 3.42 wide. Eye sizes and interdistances: AME 0.20, ALE 0.20, PME 0.16, PLE 0.18; AME–AME 0.16, AME–ALE 0.13, PME–PME 0.34, PME–PLE 0.29, AME–PME 0.23, ALE–PLE 0.17. Ocular area with white hairs. Endites reddish-brown. Leg spination: femora I pl 2 do 3 rl 2, II–III pl 3 do 3 rl 3, IV pl 1 do 3 rl 3; tibiae I–II plv 3 rlv 3, III pl 2 rl 2 plv 3 rlv 3, IV pl 3 rl 3 plv 3 rlv 3; metatarsi I–II plv 1 rlv 1, III–IV pl 4 rl 4 plv 3 rlv 3. Palp and leg measurements: palp 3.97 (1.29, 0.74, 0.76, -, 1.18), I 10.32 (2.97, 1.68, 2.51, 1.93, 1.23), II 10.31 (3.06, 1.63, 2.48, 1.99, 1.15), III 9.55 (2.76, 1.43, 2.04, 2.28, 1.04), IV 12.30 (3.34, 1.62, 2.95, 3.20, 1.19). Leg formula: 4123. Other characters same as holotype.

***Epigyne*** (Fig. 4A, B). Epigynal field with two large spots. Epigynal plate sclerotized, anteriorly to medially with narrow membranous area, laterally with large crescent-shaped sclerites. Copulatory openings hidden under epigynal plate. Copulatory ducts long and curved, subdistally to distally bifurcate. Glandular appendages globular, originating subdistally to copulatory ducts. Spermathecae large, kidney-shaped, separated by less than half of their diameter. Fertilization ducts almost as long as diameter of spermathecae, close to each other distally, pointing anteriorly.

##### Variation.

Second paratype female: total body length 10.70, carapace 5.25 long, 3.52 wide, opisthosoma 5.45 long, 3.21 wide.

##### Distribution.

China (Yunnan, type locality; Fig. 12).

#### ﻿Genus Xantharia

Taxon classificationAnimaliaAraneaeLiocranidae

Deeleman-Reinhold, 2001

D7A2B085-B6BB-53DC-8EE1-725B329B6E79

##### Type species.

*Xanthariafloreni* Deeleman-Reinhold, 2001 from Malaysia.

##### Composition.

*Xantharia* is endemic to Southeast Asia, and three species are currently included: *X.floreni* Deeleman-Reinhold, 2001 (♂♀) from Malaysia, *X.galea* Zhang, Zhang & Fu, 2010 (♂♀) from China, and *X.murphyi* Deeleman-Reinhold, 2001 (♂) from Indonesia.

##### Diagnosis.

**Figure 6.**
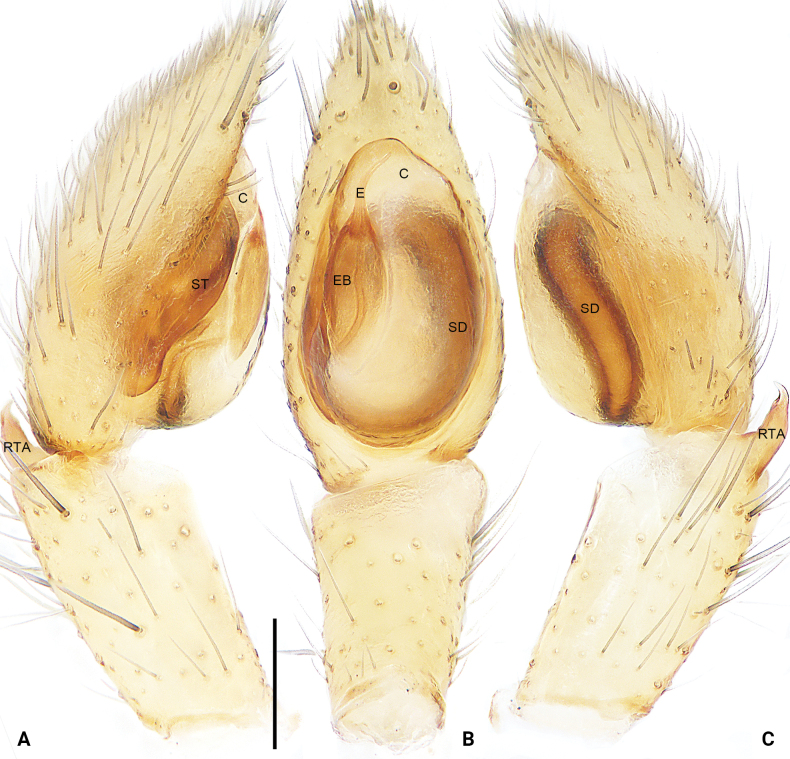
*Xanthariabaizilongi* sp. nov., holotype male (**A–C)** palp **A** prolateral view **B** ventral view **C** retrolateral view. Abbreviations: C = conductor, E = embolus, EB = embolus base, RTA = retrolateral tibial apophysis, SD = sperm duct, ST = subtegulum. Scale bar: 0.20 mm.

**Figure 7.**
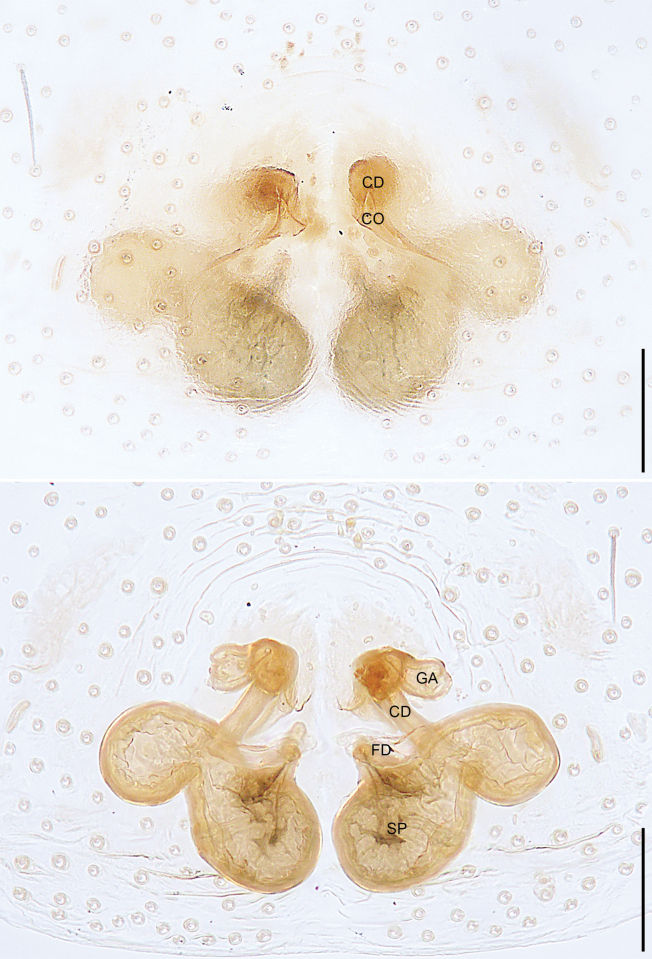
*Xanthariabaizilongi* sp. nov., paratype female (**A, B**) **A** epigyne, ventral view **B** vulva, dorsal view. Abbreviations: CD = copulatory duct, CO = copulatory opening, FD = fertilization duct, GA = glandular appendage, SP = spermathecae. Scale bars: 0.10 mm.

**Figure 8.**
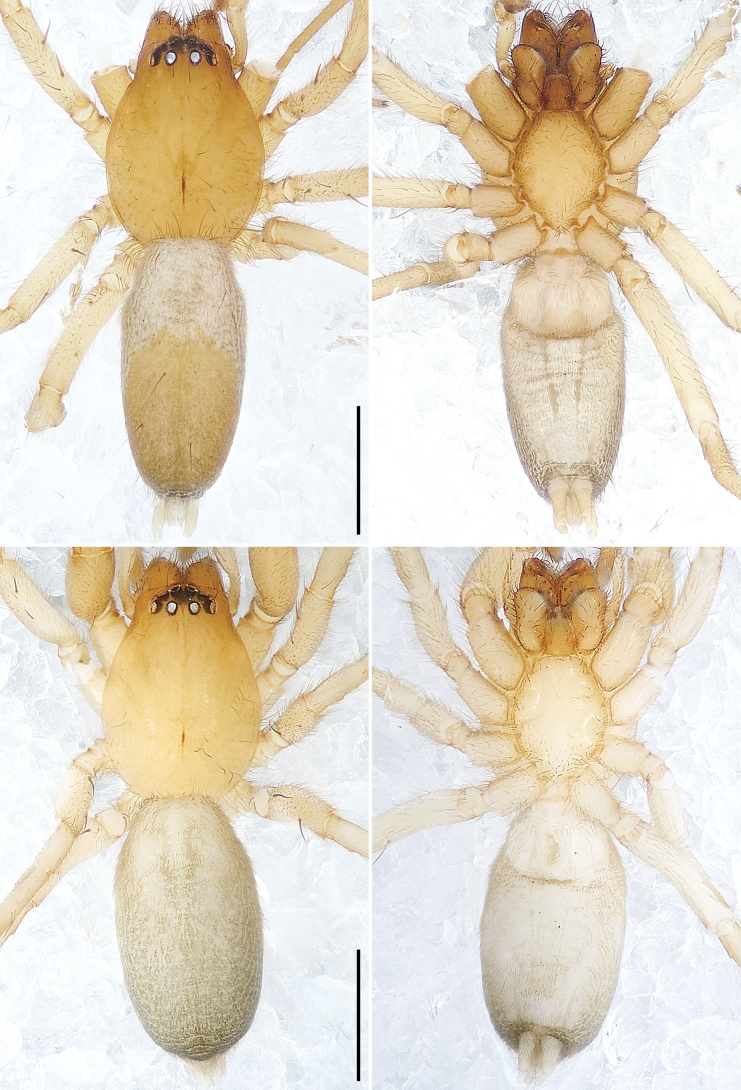
*Xanthariabaizilongi* sp. nov., holotype male (**A, B**) and paratype female (**C, D**) **A–D** habitus **A** dorsal view **B** ventral view **C** dorsal view **D** ventral view. Scale bars: 1.00 mm.

**Figure 9.**
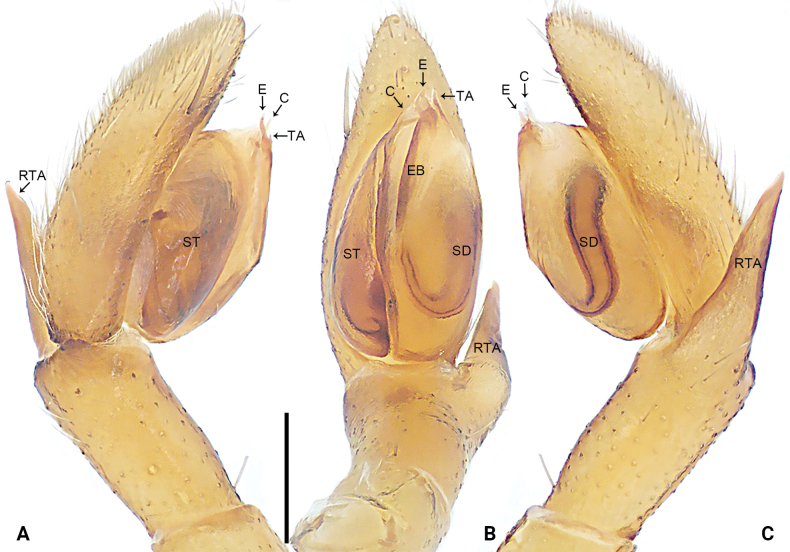
*Xanthariacucphuong* sp. nov., holotype male (**A–C)** palp **A** prolateral view **B** ventral view **C** retrolateral view. Abbreviations: C = conductor, E = embolus, EB = embolus base, RTA = retrolateral tibial apophysis, SD = sperm duct, ST = subtegulum, TA = tegular apophysis. Scale bar: 0.50 mm.

**Figure 10.**
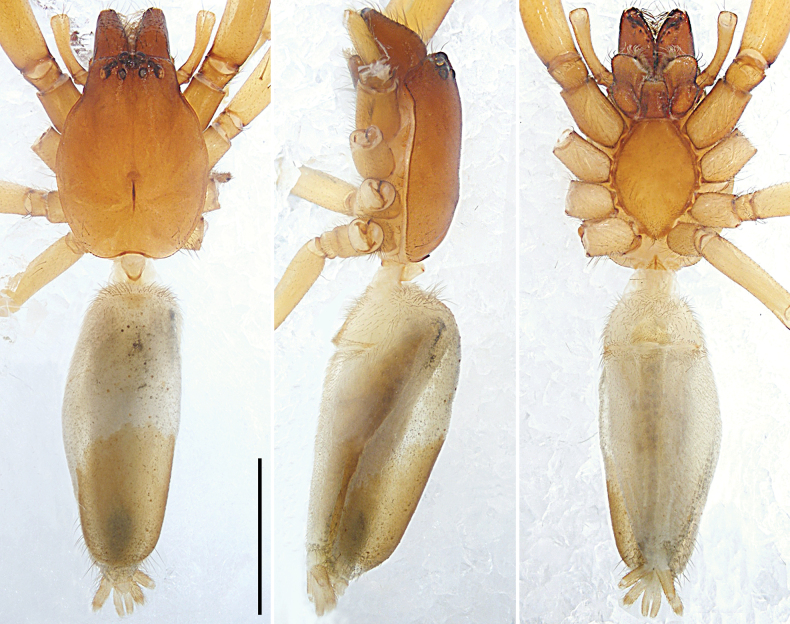
*Xanthariacucphuong* sp. nov., holotype male (**A–C)** habitus **A** dorsal view **B** lateral view **C** ventral view. Scale bar: 2.00 mm.

The genus resembles *Arabelia* Bosselaers, 2009 as the males (cf. Figs 6, 9 and Bosmans 2011: 20, figs 15, 16) have a similar wide and nearly elliptical embolic base (Figs 6B, 9B), a membranous conductor (Figs 6A–C, 9A–C), a long looping sperm duct (Figs 6A–C, 9A–C), a retrolateral tibial apophysis (Figs 6A–C, 9A–C) and females have similar globular secondary spermathecae (Fig. 7B), but can be distinguished by the endites with a diagonal depression in the middle (Figs 8B, D, 10C; absent in *Arabelia*), by the legs I distinctly stouter than legs II–IV (Figs 8A–D, 10A–C; vs. legs strength uniform in *Arabelia*), by the anterior tibiae and metatarsi spineless (Figs 8A–D, 10A–C; present in *Arabelia*), by the copulatory openings small (Fig. 7A; vs. copulatory openings large in *Arabelia*), and by the fertilization ducts originating medially (Fig. 7B; vs. fertilization ducts originating posteriorly in *Arabelia*). The genus also resembles *Drassinella* Banks, 1904 as the males have similar shape and position of embolus and sperm duct (Figs 6A–C, 9A–C), membranous conductor (Figs 6A–C, 9A–C) and retrolateral tibial apophysis (Figs 6A–C, 9A–C), but can be distinguished by the legs I distinctly stouter than legs II–IV (Figs 8A–D, 10A–C; vs. legs strength uniform in *Drassinella*), by the anterior tibiae and metatarsi spineless (Figs 8A–D, 10A–C; present in *Drassinella*), by the palpal femur without apophysis (Figs 8A, B, 10A–C; vs. palpal femur with retroventral apophysis, surface with tiny denticles in *Drassinella*), by the epigynal field with or without anterior hood (Fig. 7A; vs. epigynal field with indistinct anterior ridge in *Drassinella*), and by the fertilization ducts originating medially (Fig. 7B; vs. fertilization ducts originating posteriorly in *Drassinella*).

##### Description.

See Deeleman-Reinhold (2001).

##### Discussion.

**Figure 11.**
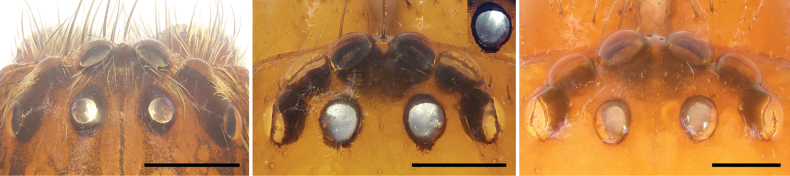
Ocular areas and tapeta of liocranids. **A***Sinocranummenghai* sp. nov. **B***Xanthariabaizilongi* sp. nov., inset shows oblique tapetum of PME **C***X.cucphuong* sp. nov. Scale bars: 0.50 mm (**A**); 0.20 mm (**B, C**).

*Xantharia* is placed in Liocranidae based on the following combination of characters: posterior median eye tapeta forming 90° angle (Ramírez 2014), endites with a diagonal depression in the middle like *Drassinella* Banks, 1904 and *Jacaena* Thorell, 1897 (e.g., Platnick and Ubick 1989; fig. 778 in Deeleman-Reinhold 2001; fig. 1C in Liu et al. 2020), anterior tibiae and metatarsi spineless, like in *Sphingius* Thorell, 1890 (e.g., Zhang et al. 2009), shape and position of embolus and sperm duct like *Arabelia* Bosselaers, 2009 and *Drassinella* (e.g., figs 1, 5 in Platnick and Ubick 1989; fig. 3D in Mu and Zhang 2022), most of the epigynal field anteriorly hood like *Arabelia*, *Mesiotelus* Simon, 1897 and *Sphingius* (e.g., figs 18, 27 in Zhang et al. 2009; fig. 4A in Mu and Zhang 2022; figs 4–7, 15–18 in Coşar et al. 2023). At the same time, *Xantharia* of Liocranidae can be distinguished from Miturgidae by the posterior median eye tapeta forming 90° angle (Fig. 11A–C), but grate-shaped in Miturgidae (e.g., fig. 14a in Raven 2009), eight eyes in two rows, AER slightly recurved, PER almost straight in dorsal view (Fig. 11A–C), but PER slightly procurved to recurved in Miturgidae (e.g., fig. 1c in Raven 2009), cymbium without retrolateral groove (Figs 6C, 9C), but present in Miturgidae (e.g., figs 140B, 145C–F in Ramírez 2014; figs 2D, 3D–E in Sankaran and Sebastian 2019; figs 1C, 2C in Sánchez-Ruiz et al. 2020), RTA without canal and membranous area (Figs 6A–C, 9A–C), but present in Miturgidae (e.g., figs 6c, 14e in Raven 2009; fig. 146B in Ramírez 2014). The genus can also be distinguished from Clubionidae in Asia by the posterior median eye tapeta forming 90° angle (Fig. 11A–C), but absent in Clubionidae (Ramírez 2014), endites with diagonal depression in the middle (Figs 8B, D, 10C), but absent in Clubionidae (e.g., figs 1G, 2G in Zhang et al. 2021a; figs 2H, 4H in Zhang et al. 2021b; figs 2H, 12H in Zhang et al. 2021c), ocular area covering three-fifths of the anterior width of the carapace (Figs 8A, C, 10A), but four-fifths in Clubionidae (e.g., figs 1F, 2F in Zhang et al. 2021a; figs 2E, 4E in Zhang et al. 2021b; figs 2E, 8E in Zhang et al. 2021c), wall of the primary spermathecae and secondary spermathecae almost uniform (Fig. 7B), but bursae thin-walled and spermathecae thick-walled in Clubionidae (e.g., figs 6D, 8D in Zhang et al. 2021a; figs 2D, 4D in Zhang et al. 2021b; figs 14D, 16D in Zhang et al. 2021c).

#### ﻿ Xantharia baizilongi

Taxon classificationAnimaliaAraneaeLiocranidae

Chu & Li sp. nov.

54FFD670-08A8-5B84-9CAE-5E28EF9B18A3

https://zoobank.org/8C40E936-5033-4547-B6A0-9CCEFB91AD51

Figs 6 , 7 , 8 , 11B

##### Type material.

***Holotype***: 1♂ (IZCAS-Ar44621), **China**, Yunnan, Xishuangbanna, Mengla County, Menglun Town, Xishuangbanna Tropical Botanical Garden, 21°53.886′N, 101°16.719′E, 568 m, hand catch in leaf litter, 12 May 2019, Z. Bai leg. ***Paratypes***: 1♂ (IZCAS-Ar44622) and 2♀ (IZCAS-Ar44623, 44624), same data as holotype.

##### Etymology.

The specific name is a patronym in honour of the collector Zilong Bai; noun (name) in genitive case.

##### Diagnosis.

The new species resembles *X.floreni* Deeleman-Reinhold, 2001 (cf. Figs 6–8, 11B and Deeleman-Reinhold 2001: 217, figs 235, 246, 247, 257–263) as the males have a similar long looping sperm duct (Fig. 6A–C), wide and elliptical embolic base (Fig. 6B) and females have similar laminar fertilization ducts (Fig. 7B). Males can be distinguished by the embolic tip not exceeding bulb distally (Fig. 6B; present), by the palp with conductor without tegular apophysis (Fig. 6A–C; vs. palp with indistinct conductor and tegular apophysis), and by the retrolateral tibial apophysis arising from rear part of the tibia distally, invisible in ventral view (Fig. 6A–C; vs. retrolateral tibial apophysis arising from middle part of tibia distally, visible in ventral view). Females can be distinguished by the epigyne without anterior hood (Fig. 7A; present), by the vulva with glandular appendages (Fig. 7B; absent), by the primary spermathecae and secondary spermathecae connected to each other (Fig. 7B; vs. primary spermathecae and secondary spermathecae separated from each other), and by the primary spermathecae larger than secondary spermathecae (Fig. 7B; vs. primary spermathecae smaller than secondary spermathecae). This species also resembles *X.galea* Zhang, Zhang & Fu, 2010 (cf. Figs 6–8, 11B and Zhang et al. 2010: 66, figs 1–11) as the males have a similar long looping sperm duct (Fig. 6A–C), wide and elliptical embolic base (Fig. 6B), membranous conductor (Fig. 6A–C), and females have similar tubular copulatory ducts (Fig. 7B) and laminar fertilization ducts (Fig. 7B). Males can be distinguished by the embolus originating 8:00 o’clock, embolic tip not exceeding bulb distally, with slight retrolateral curvature (Fig. 6B; vs. embolus originating 9:00 o’clock, embolic tip exceeding bulb distally, with slight prolateral curvature), and by the long retrolateral tibial apophysis (Fig. 6A–C; vs. short retrolateral tibial apophysis). Females can be distinguished by the epigyne without an anterior hood (Fig. 7A; present), by the copulatory openings triangular (Fig. 7A; vs. copulatory openings circular), by the vulva with glandular appendages (Fig. 7B; absent), by the primary spermathecae and secondary spermathecae connected to each other (Fig. 7B; vs. primary spermathecae and secondary spermathecae separated and with common horizontal plane), and by the primary spermathecae larger than secondary spermathecae (Fig. 7B; vs. primary spermathecae smaller than secondary spermathecae).

##### Description.

**Male** (holotype; Figs 8A, B, 11B). Total body length 3.62, carapace 1.65 long, 1.23 wide, opisthosoma 1.97 long, 0.95 wide. Eye sizes and interdistances: AME 0.09, ALE 0.11, PME 0.08, PLE 0.10; AME–AME 0.06, AME–ALE 0.02, PME–PME 0.11, PME–PLE 0.09, AME–PME 0.09, ALE–PLE 0.04. Carapace yellowish-brown without pattern, pear-shaped; fovea reddish-brown. Chelicerae yellowish-brown, with several setae on anterior surface, with two promarginal and two retromarginal teeth. Endites yellowish-brown, longer than wide, widest anteriorly, concave laterally, with diagonal depression in middle, subapically with semicircular membranous area and dense scopula. Labium reddish-brown, nearly isosceles trapezoidal, with constriction subbasally and sparse scopula apically. Sternum yellow without pattern, margin yellowish-brown, narrowing anteriorly, with precoxal triangles and intercoxal extensions. Legs yellowish without pattern; legs I distinctly darker and stouter than legs II–IV. Leg spination: femora II pl 2, III–IV do 2; tibiae III–IV pl 1; metatarsi III–IV pl 1 rl 1 plv 2 rlv 2. Palp and leg measurements: palp 1.49 (0.56, 0.21, 0.29, -, 0.43), I 4.44 (1.22, 0.78, 1.04, 0.98, 0.42), II 3.73 (1.09, 0.62, 0.81, 0.82, 0.39), III 3.35 (0.95, 0.50, 0.65, 0.87, 0.38), IV 4.46 (1.28, 0.63, 0.96, 1.16, 0.43). Leg formula: 4123. Dorsal opisthosoma grey, posteriorly yellow occupying more than half of dorsal surface, with dark margin around spinnerets. Lateral opisthosoma with dark stripes. Ventral opisthosoma grey with dark margin around spinnerets. Spinnerets yellowish.

***Palp*** (Fig. 6A–C). Retrolateral tibial apophysis short, curvature distally, with wide base and narrow tip. Bulb oval, subtegulum sclerotized, visible in ventral view; sperm duct distinct, running around tegulum. Embolus originating at 8:00 o’clock, embolic base sclerotized, wide and elliptical; embolic tip membranous, situated distally at 12:00 o’clock. Conductor membranous, nearly fan-shaped, originating distally to bulb.

**Female** (paratype; Fig. 8C, D). Total body length 3.71, carapace 1.69 long, 1.23 wide, opisthosoma 2.02 long, 1.11 wide. Eye sizes and interdistances: AME 0.09, ALE 0.10, PME 0.08, PLE 0.11; AME–AME 0.06, AME–ALE 0.02, PME–PME 0.11, PME–PLE 0.08, AME–PME 0.09, ALE–PLE 0.04. Carapace yellowish without pattern, ocular area yellowish-brown; fovea shorter. Chelicerae, endites and labium yellowish; endites with indistinct diagonal depression in middle. Sternum without distinct precoxal triangles. Leg spination: femora II–IV do 2; tibiae III–IV pl 1; metatarsi III–IV pl 1 rl 1 plv 2 rlv 2. Palp and leg measurements: palp 1.51 (0.57, 0.24, 0.29, -, 0.41), I 4.72 (1.30, 0.78, 1.13, 1.03, 0.48), II 4.07 (1.14, 0.63, 0.88, 0.97, 0.45), III 3.73 (1.04, 0.55, 0.72, 1.01, 0.41), IV 4.91 (1.40, 0.65, 1.13, 1.25, 0.48). Leg formula: 4123. Dorsal opisthosoma grey with dark spots. Other characters same as holotype.

***Epigyne*** (Fig. 7A, B). Epigynal plate simple; copulatory openings triangular, originating centrally to epigynal field. Copulatory ducts long, with sharp twist at its base, presenting spherical. Glandular appendages round. Primary spermathecae large, elliptical, almost adjacent to each other; secondary spermathecae small, nearly globular, separated by more than their diameter; primary spermathecae and secondary spermathecae connected to each other. Fertilization ducts originating anteriorly to primary spermathecae, pointing laterally.

##### Variation.

Paratype male: total body length 3.33, carapace 1.58 long, 1.14 wide, opisthosoma 1.75 long, 1.04 wide. Second paratype female: total body length 4.30, carapace 1.80 long, 1.38 wide, opisthosoma 2.50 long, 1.24 wide.

##### Distribution.

**Figure 12.**
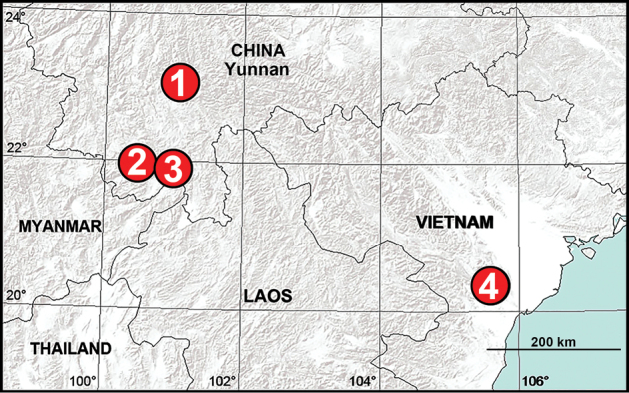
New distribution records of liocranid spiders from China and Vietnam **1***Koppeninger* sp. nov. **2***Sinocranummenghai* sp. nov. **3***Xanthariabaizilongi* sp. nov. **4***X.cucphuong* sp. nov.

China (Yunnan, type locality; Fig. 12).

#### ﻿ Xantharia cucphuong

Taxon classificationAnimaliaAraneaeLiocranidae

Chu & Li sp. nov.

84B6958A-C2C1-58F3-BE4F-4136E0666FB6

https://zoobank.org/51250F06-BAF5-4F21-BC76-F156931FB94C

Figs 9 , 10 , 11C

##### Type material.

***Holotype***: 1♂ (IZCAS-Ar44625), **Vietnam**, Ninh Binh, Cuc Phuong National Park, 20°20.568′N, 105°36.024′E, 408 m, hand catch in leaf litter, 8 October 2007, D.S. Pham leg.

##### Etymology.

The specific name refers to the type locality and is a noun in apposition.

##### Diagnosis.

The new species resembles *X.galea* Zhang, Zhang & Fu, 2010 (cf. Figs 9, 10, 11C and Zhang et al. 2010: 66, figs 1–11) by the similar membranous conductor (Fig. 9A–C) and sclerotized subtegulum (Fig. 9A, B). Males can be distinguished by the embolic tip membranous, wide, nearly quadrangular (Fig. 9A–C; vs. embolic tip sclerotized, thin and filiform), by the palp with tegular apophysis (Fig. 9A–C; absent), by the sperm duct separated from the base of tegulum by nearly double the width of the sperm duct (Fig. 9B; vs. sperm duct extending to the base of tegulum), and by the retrolateral tibial apophysis long and straight (Fig. 9A–C; vs. retrolateral tibial apophysis short and beak-shaped retrolaterally). Female unknown.

##### Description.

**Male** (holotype; Figs 10A–C, 11C). Total body length 6.73, carapace 2.69 long, 1.95 wide, opisthosoma 4.04 long, 1.49 wide. Eye sizes and interdistances: AME 0.13, ALE 0.14, PME 0.10, PLE 0.13; AME–AME 0.07, AME–ALE 0.03, PME–PME 0.18, PME–PLE 0.15, AME–PME 0.12, ALE–PLE 0.07. Carapace reddish-brown, darker in ocular area, without pattern; fovea reddish-brown. Chelicerae reddish-brown, with several setae on anterior surface, with two promarginal and two retromarginal teeth. Endites reddish-brown, longer than wide, widest anteriorly, concave laterally, with diagonal depression in middle, subapically with semicircular membranous area and dense scopula. Labium reddish-brown, nearly isosceles trapezoidal, with constriction subbasally and scopula apically. Sternum reddish-brown without pattern, narrowing anteriorly, with precoxal triangles and intercoxal extensions. Legs yellowish-brown without pattern; legs I distinctly darker and stouter than legs II–IV. Leg spination: femora II pl 2 do 1, III pl 1 do 1 rl 1, IV do 2; tibia III pl 2 rl 1 rlv 2; metatarsus III pl 2 rl 2 plv 2 rlv 2. Palp and leg measurements: palp 2.52 (0.92, 0.40, 0.43, -, 0.77), I 7.31 (2.04, 1.17, 1.74, 1.68, 0.68), II 6.55 (1.83, 0.99, 1.51, 1.58, 0.64), III 5.74 (1.65, 0.67, 1.34, 1.39, 0.69), IV - (1.77, -, -, -, -). Dorsal opisthosoma grey, posteriorly yellow occupying about half of dorsal surface, with dark patterns. Lateral opisthosoma with dark spots. Ventral opisthosoma grey with dark margin around spinnerets. Spinnerets yellow.

***Palp*** (Fig. 9A–C). Retrolateral tibial apophysis long and straight, with wide base and narrow, blunt tip. Bulb oval, subtegulum sclerotized, visible in ventral view; sperm duct distinct, separated from the base of tegulum by nearly double the width of the sperm duct. Embolus originating 9:00 o’clock, embolic base wide and elliptical, sclerotized along margin; embolic tip membranous, nearly quadrangular, situated distally. Conductor membranous, nearly fan-shaped, originating distally to bulb. Tegular apophysis membranous and triangular, weakly sclerotized along margin.

##### Distribution.

Vietnam (Ninh Binh, type locality; Fig. 12).

## Supplementary Material

XML Treatment for Koppe

XML Treatment for Koppe ninger

XML Treatment for Sinocranum

XML Treatment for Sinocranum menghai

XML Treatment for Xantharia

XML Treatment for Xantharia baizilongi

XML Treatment for Xantharia cucphuong

## ﻿Additional information

### Author ORCIDs

Chang Chu https://orcid.org/0000-0003-3520-5463

Shuqiang Li https://orcid.org/0000-0002-3290-5416

Yanbin Yao https://orcid.org/0000-0002-2560-9299

Zhiyuan Yao https://orcid.org/0000-0002-1631-0949

### Data availability

All of the data that support the findings of this study are available in the main text.
